# Magnetic Nanoparticle-Reduced Graphene Oxide Nanocomposite as a Novel Bioelectrode for Mediatorless-Membraneless Glucose Enzymatic Biofuel Cells

**DOI:** 10.1038/s41598-017-12417-0

**Published:** 2017-10-10

**Authors:** Saithip Pakapongpan, Adisorn Tuantranont, Rungtiva P. Poo-arporn

**Affiliations:** 1 Biological Engineering Program, Faculty of Engineering, King Mongkut’s University of Technology Thonburi, Bangkok, 10140 Thailand; 2 Thailand Organic and Printed Electronics Innovation Center, National Electronics and Computer Technology Center, NSTDA, Pathum Thani, 12120 Thailand

## Abstract

In this work, an enzymatic biofuel cell (EBC) based on a membraneless and mediatorless glucose enzymatic fuel cell system was constructed for operation in physiological conditions (pH 7.0 and temperature 37 °C). The new platform EBC made of nanocomposite, including magnetic nanoparticles (Fe_3_O_4_ NPs) and reduced graphene oxide (RGO), was used for the immobilization of glucose oxidase (GOD) as bioanode and bilirubin oxidase (BOD) as biocathode. The EBC bioelectrodes were fabricated without binder or adhesive agents for immobilized enzyme and the first EBC using superparamagnetic properties with Fe_3_O_4_ NPs has been reported. The performance of the EBC was evaluated with promising results. In EBC tests, the maximum power density of the EBC was 73.7 μW cm^−2^ and an open circuit voltage (OCV) as +0.63 V with 5 mM of glucose concentration for the physiological condition of humans. The Fe_3_O_4_-RGO nanocomposite offers remarkable enhancement in large surface areas, is a favorable environment for enzyme immobilization, and facilitates electron transfer between enzymes and electrode surfaces. Fe_3_O_4_ and RGO have been implied as new promising composite nanomaterials for immobilizing enzymes and efficient platforms due to their superparamagnetism properties. Thus, glucose EBCs could potentially be used as self-powered biosensors or electric power sources for biomedical device applications.

## Introduction

An enzymatic biofuel cell (EBC) is a tool used to generate electrical energy from fuels in combination with dioxygen, which is based on the transformation of chemical energy directly into electricity via redox reactions or chemical reactions without going through the combustion process, instead utilizing natural enzymes as the catalyst^1,2^. They are useful for alternative power sources and for *in vivo* applications such as implantable biomedical devices, miniaturized sensors transmitters and artificial organs^3,4^. EBCs are of interest as power sources for biosensors and medical implants (e.g. insulin pumps, cardiac pacemakers, and other devices). To be able to activate commonly-used microelectronic devices (such as commercial pacemakers), appropriate output voltages (minimum of 1.4 V) are required^5^. Glucose biofuel cells are ideally suited for implantable applications. The possibility to generate electrical power via living organisms directed biofuel cell research since the two required compounds (glucose and oxygen) are present in body fluids. To date, the vast majority of glucose biofuel cells are based on the enzymatic oxidation of glucose as a fuel at the bioanode and oxygen reduction at the biocathode. Usually, enzyme immobilization occurs by either chemical or physical means such as covalent binding^6^, cross-linking^7^, sol–gel^8^, mesoporus^9^ and entrapment in polymer^10^. However, the majority of EBC systems lack an efficient enzyme immobilization technique, so the production of electrical energy is not adequate for the device application. The enzyme on the electrode surface usually does not achieve significant electron transfer between the immobilized enzymes and the current collector or electrode. To solve this problem, significant efforts have been made to improve the direct electron transfer (DET) or mediated electron transfer (MET) reactions of the enzymes by immobilizing onto various interfaces or by using redox mediators. The publications of research generally utilize membrane and toxic mediators to improve performance, which is not appropriate for cases of implanted devices in the human body. Recently, DET-based EBC and mediatorless EBC have become more interesting due to the elimination of the mediator leak issue. Nevertheless, the rate of electron transfer between the redox center of biocatalysts and the underlying electrodes is very slow because of the electrical insulation of the active sites of the enzyme by the surrounding protein shells, leading to the low efficiency and small power output of EBCs. Moreover, the leaching out of enzymes is still a limiting factor for constructing an applicable EBC. In order to improve the electron transfer rate between the enzyme and electrode surface, some of the new trends in catalyst design for biofuel cells include the incorporation of nanoscale materials and metallic nanoparticles into the bioelectrode structure. The use of nanomaterials to develop biofuel cell electrodes has generated unprecedented interest due to their high surface area for enzyme immobilization, favorable electronic properties and electrocatalytic activity. The various structures of nanomaterials employed to modify electrodes, such as carbon nanotubes (CNT)^11^, mesoporus carbon^12^ and gold nanoparticles (AuNPs)^13^, as well as polyaniline nanofibers^14^, can increase electrical output and stability, in addition to improving electron transfer at the working electrode. Among nanomaterials, carbon-based nanomaterials and nanocomposites can provide the enhanced performance properties of electrodes such as CNT-Platinum nanoparticles^15^, CNT-dendrimer^16^ and CNT-polypyrrole^17^, 3D graphene-SWCNT hybrid^18^. Li *et al*. were the first to report EBC use of graphene sheet/enzyme composites to improve electron transfer and electricity generation^8^. Even though these combinations can increase the electrical output and stability as well as improve the electron transfer at working electrode, the mediator and membrane were still applied for the EBC. Graphene is a single layer of carbon atoms with a two-dimensional honeycomb sp2 carbon lattice. It possesses many unique features such as large surface area, good electrical conductivity, and thermal as well as mechanical properties^19^. The suitable matrix with good electrical conductivity, stability, and antifouling property to immobilize enzymes at the electrode surface is one of the most important tasks in the fabrication of mediatorless glucose EBC. Well-established magnetic nanoparticles with appropriate surface chemistry have been widely used in pollution remediation. Magnetic nanoparticles (Fe_3_O_4_ NPs) have special properties such as good biocompatibility, strong superparamagnetics, low toxicity, large surface-to-volume ratio, high surface reaction activity, and strong adsorption ability to immobilize desired biomolecules. It also uses an easy preparation process^20–22^. Fe_3_O_4_ NPs formed on the surface of graphene provide more surface area for enzymes and good environmental biocompatibility for enzymes immobilized. Herein, a novel bioelectrode approach was designed and developed for glucose EBC by using nanocomposites of Fe_3_O_4_ NPs, RGO and a group of enzymes, including glucose oxidase and bilirubin oxidase to increase the immobilization of the enzyme, improve the direct electron transfer at the electrode, and improve the stability of the electrode without a binder or membrane while preventing enzyme leaching. Fe_3_O_4_-RGO nanocomposite was prepared by covalent bond and immobilized enzymes with electrostatic interaction. As well, the immobilization of enzymes and mediators usually involves complicated procedures. This work provides a simple preparation of bioelectrodes, RGO-Fe_3_O_4_ nanocomposite could be used to attracted enzymes onto electrode surfaces with a strong magnetic force. However, the use of Fe_3_O_4_ NPs in biofuel cells has not been reported. As such, the electrochemical performance of glucose biofuel cells was investigated by using electrochemical methods. These enzymatic biofuel cells might be able to replace the batteries in medical devices and other applications in the future.

## Results and Discussion

### Characterization of Fe_3_O_4_-RGO/GOD nanocomposite

**Figure 1:**
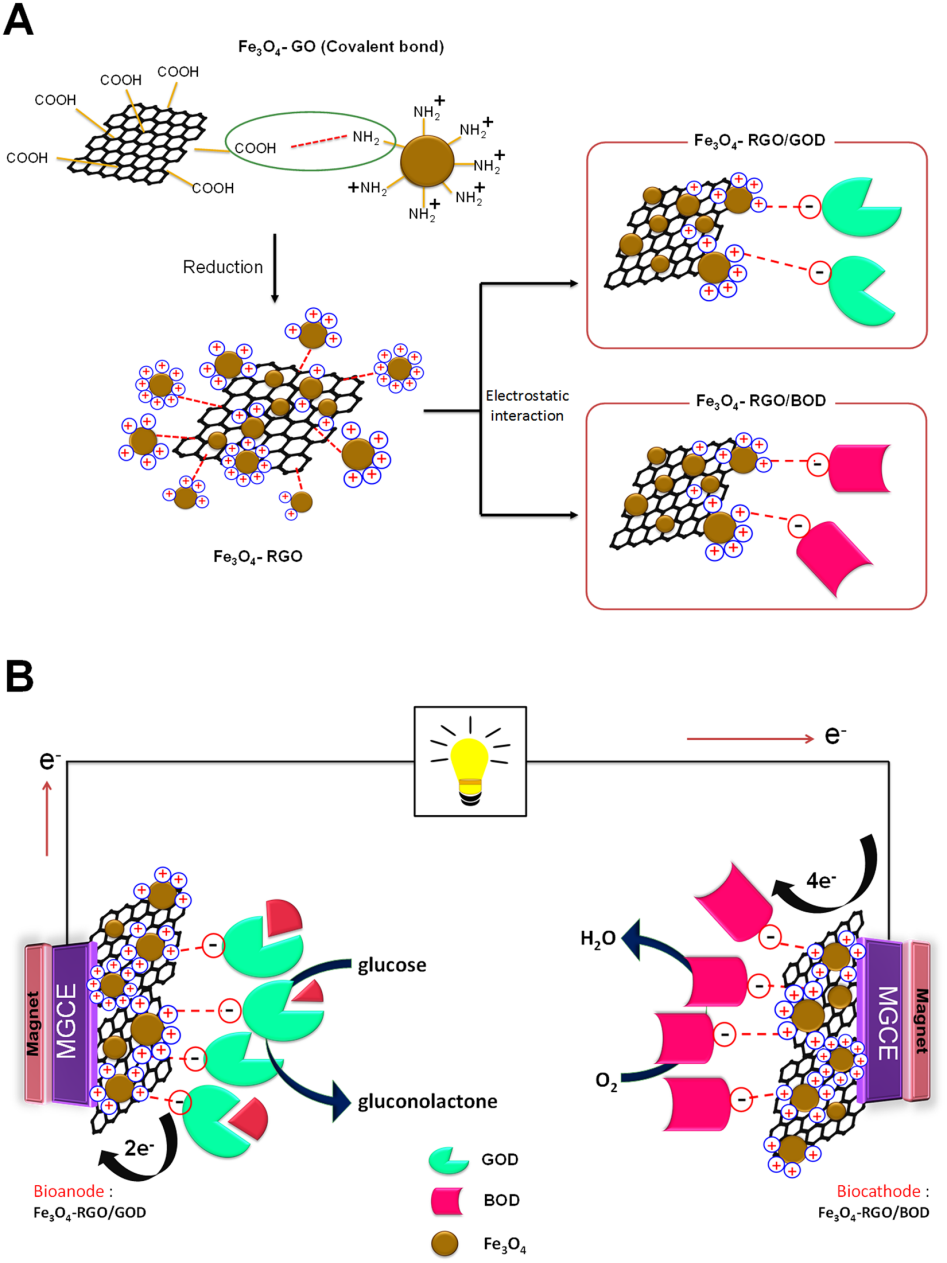
Schematic representation of (**A**) the preaparation of Fe_3_O_4_-RGO and (**B**) the fabricated bioelectrodes for glucose EBC.

**Figure 2:**
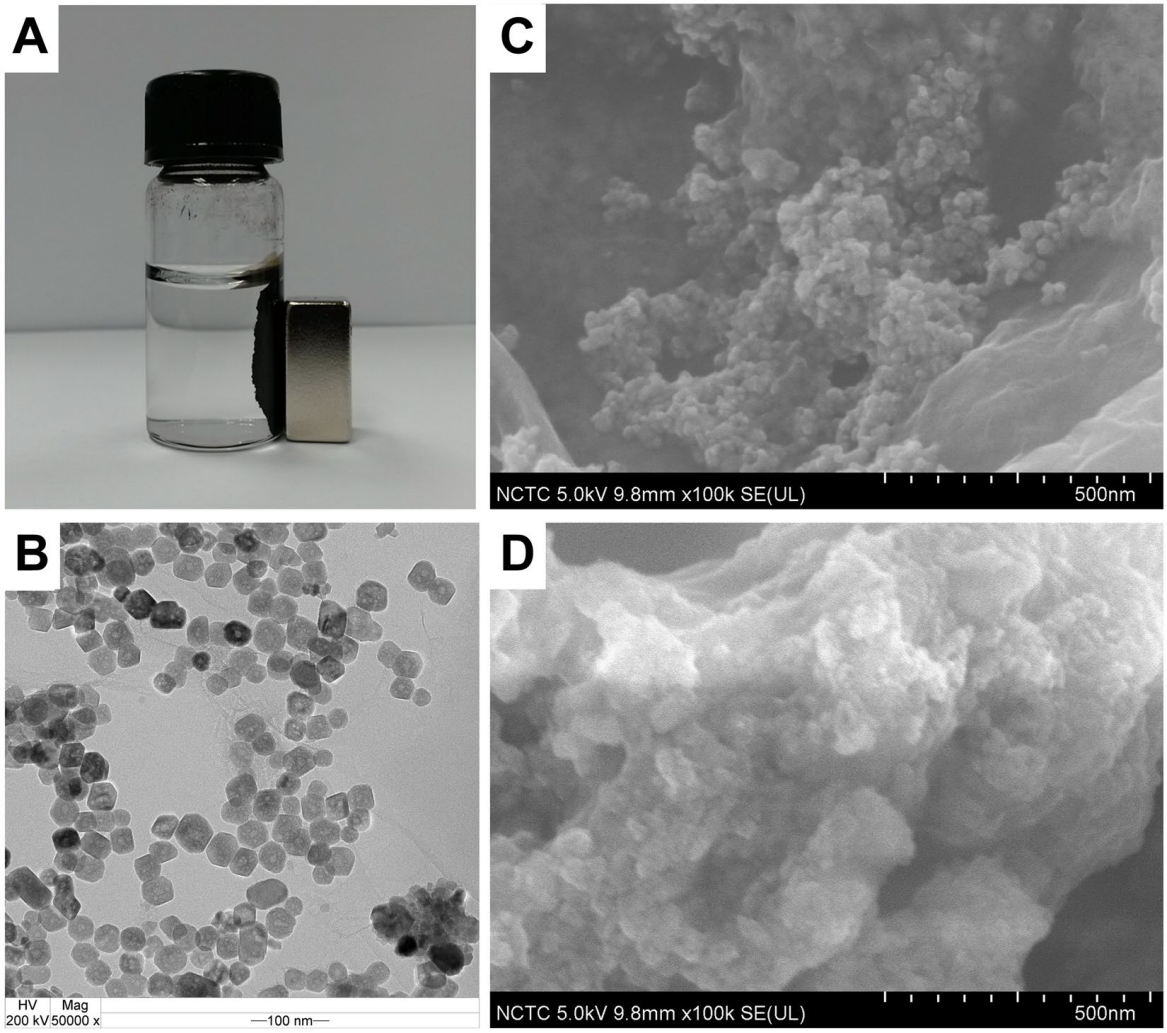
(**A**) Photograph of Fe_3_O_4_-RGO attracted by external magnet. (**B**) TEM image of Fe_3_O_4_-RGO. SEM images of (**C**) Fe_3_O_4_-RGO and (**D**) Fe_3_O_4_-RGO/GOD.

**Figure 3:**
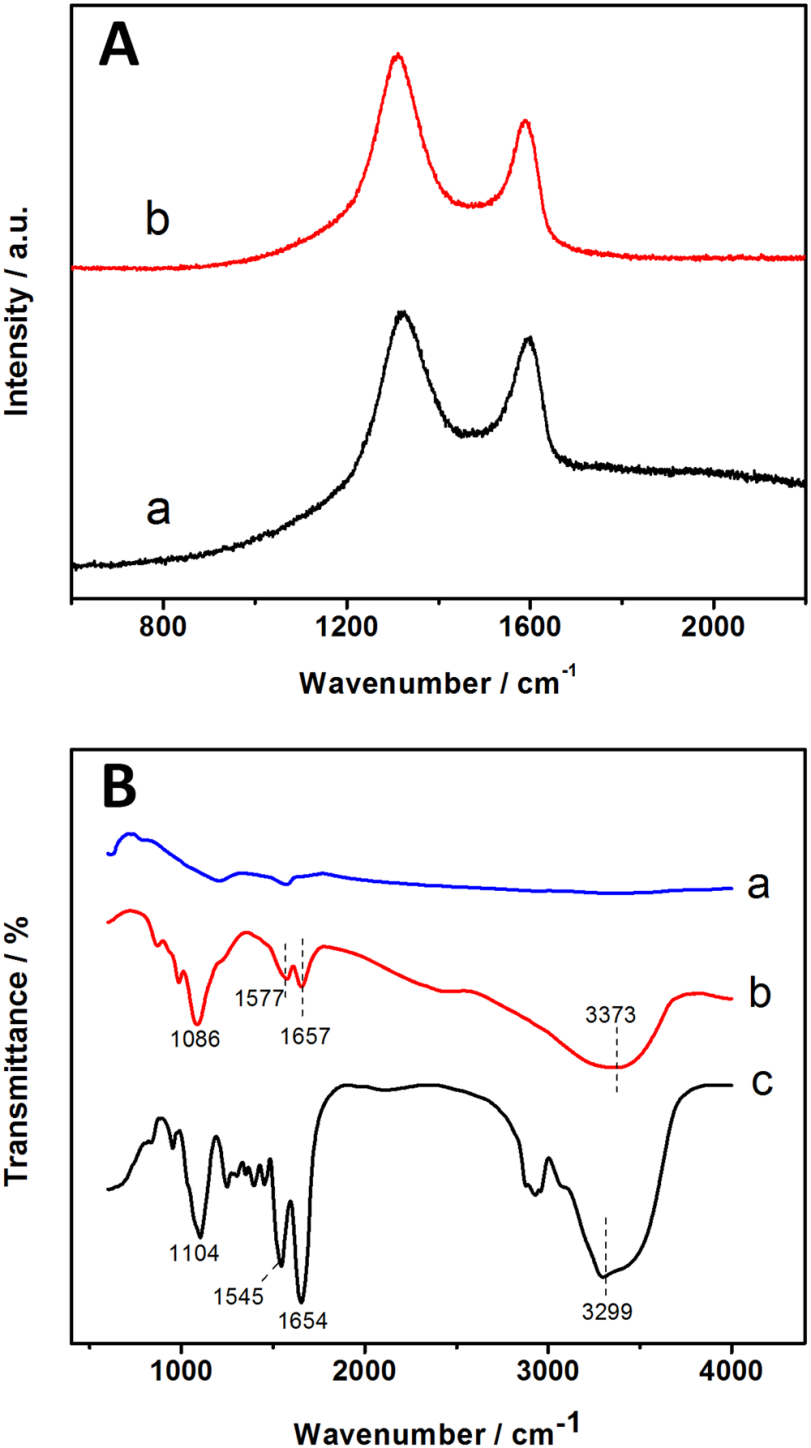
(**A**) Raman spectrum of Fe_3_O_4_-GO (a) and Fe_3_O_4_-RGO (b). (**B**) FT-IR spectrum of Fe_3_O_4_-RGO (a), Fe_3_O_4_-RGO/GOD (b) and GOD (c).

The bioelectrodes fabrication was shown as schematic in Fig. 1. Due to Fe_3_O_4_ NPs being surrounded by a positive charge, they play an important role in immobilizing enzymes through electrostatic interaction. GOD is a negatively charged biomolecule at pH 7.0^23^ that can be easily immobilized onto the positively charged amino group on Fe_3_O_4_ NPs surface via electrostatic interaction. Figure 2A, the resultant Fe_3_O_4_-RGO nanohybrids could be immediately separated from the mixture when a magnet was placed nearby the glass vial within 30 second resulting in a clear and transparent solution. Thus, the attraction and dispersion processes can be readily altered by applying and removing an external magnetic field. The morphology of Fe_3_O_4_-RGO and Fe_3_O_4_-RGO/GOD nanocomposite was characterized by transmission electron microscopy (TEM) and scanning electron microscopy (SEM). Figure 2B shows a TEM image of Fe_3_O_4_-RGO. It can be seen that Fe_3_O_4_ NPs was distributed on the RGO sheet revealing a stacked, crumpled, wrinkled and rippled structure. Particle size was estimated to be in the range of 15–32 nm. GOD was immobilized on the Fe_3_O_4_-RGO nanocomposite by electrostatic force, as shown by the SEM image in Fig. 2D, where Fe_3_O_4_-RGO/GOD appears the spherical Fe_3_O_4_ NPs were roughness and the particles size increased dramatically and is clearly observed compared with SEM image of Fe_3_O_4_-RGO in Fig. 2C. This indicated that the protein globular structure of GOD was uniform on the Fe_3_O_4_-RGO structure. Figure 3A shows the Raman spectrum of Fe_3_O_4_-RGO, which exhibited peaks at 1371 cm^−1^ and 1591 cm^−1^ that correspond to the D band of the breathing mode of k-point phonons of A1g symmetry and G-band of the first-order scattering of the E2g phonons^24^, respectively. The intensity ratios *I*_*D*_/*I*_*G*_ of Fe_3_O_4_-GO and Fe_3_O_4_-RGO were found to be 1.06 and 1.13, respectively, indicating the greater sp^2^ characteristic of graphene. This increase of *I*_*D*_/*I*_*G*_ ratio is due to the decrease of the sp^2^ in-plane domain induced by the introduction of defects and disorder of the sp^2^ domain. This indicates that sp^2^ domains of Fe_3_O_4_-GO are formed during reduction using glucose as the reducing agent. FT-IR spectra of Fe_3_O_4_-RGO, Fe_3_O_4_-RGO/GOD and GOD are presented in Fig. 3B at curve a, b and c, respectively. For the Fe_3_O_4_-RGO, no obvious absorption peak was observed. The FTIR spectrum of native GOD shows two characteristic peaks at 1654 and 1545 cm^−1^, which are attributed to amide I and II bands of protein that can provide detailed information on the secondary structure of the polypeptide chain^25,26^. The band at 1104 cm^−1^ was C–O bond stretching. The broad and strong peak at 3299 cm^−1^ was assigned to hydroxyl (OH) stretching vibrations^27^. Compared to Fe_3_O_4_-RGO in curve a, the FTIR spectrum of Fe_3_O_4_-RGO-GOD exhibited absorption peaks at 1086, 1577, 1657 and 3373 cm^−1^. The positioning was similar to that of pure GOD, suggesting enzyme GOD successfully immobilized on Fe_3_O_4_-RGO and could keep its native structure after being immobilized in the Fe_3_O_4_-RGO. Zeta potential measurement was performed under neutral conditions to verify the surface charges of Fe_3_O_4_-NH_2_, Fe_3_O_4_-GO, Fe_3_O_4_-RGO and GOD. The zeta potential (ζ) values were presented in Fig. S1. The Fe_3_O_4_-NH_2_ contain large amount of amine groups at neutral pH in DI water. The amine functionalized Fe_3_O_4_ nanoparticles showed a positive zeta potential of 30.6 mV due to the due to the protonation of its –NH_2_ group on the surface. The Fe_3_O_4_-NH_2_ was covalent chemical bond with the carboxylic group of the GO present as Fe_3_O_4_-GO. The zeta potential of −16 mV for Fe_3_O_4_-GO can be explained by the presence of bulky oxygen groups such as carboxyl groups on GO sheet. After reduction process of GO to RGO, the zeta potentials of Fe_3_O_4_-RGO was 18.5 mV due to positively charge of Fe_3_O_4_ on RGO surface. The isoelectric point (pI) of GOD is 4.2, which reveals that GOD carries net negative charges at pH 7.0. The results showed that GOD is negatively charged at pH 7.0 which corresponding to zeta potential of GOD in PBS pH 7.0 was −20.5 mV. Therefore, the negative surface charge of GOD could be easily adsorbed onto the positively charged on Fe_3_O_4_-RGO surface through the electrostatic interactions.

### Electrochemical behavior of magnetic glassy carbon electrode (MGCE)

**Figure 4:**
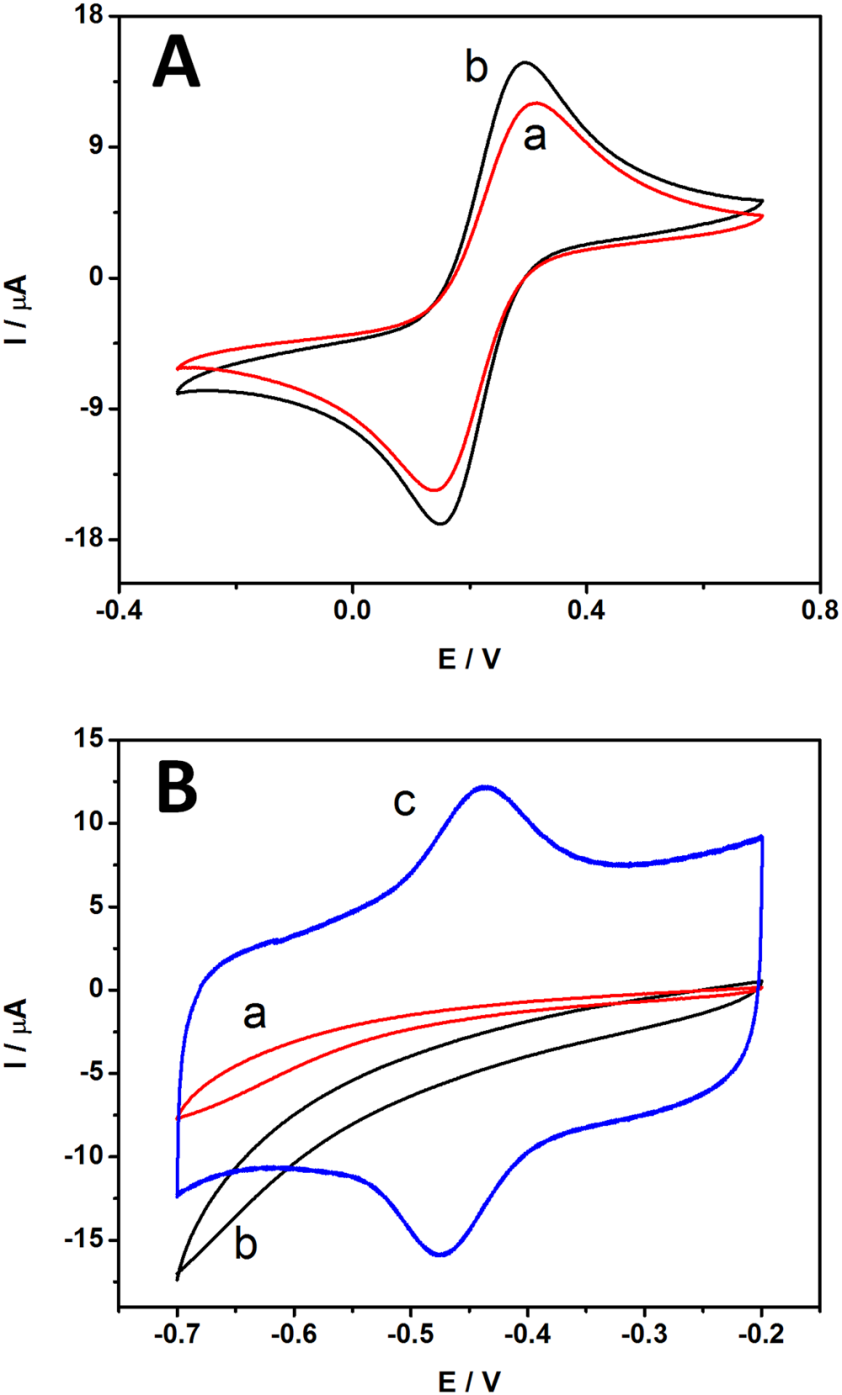
(**A**) Cyclic voltammograms (CVs) of bare GCE (a) and bare MGCE (b) in 0.1 M PBS pH 7.0 containing 2 mM K_3_Fe(CN)_6_ at a scan rate of 50 mV/s. (**B**) CVs of the different modified electrode with GOD/MGCE (a), Fe_3_O_4_/GOD/MGCE (b) and Fe_3_O_4_-RGO/GOD/MGCE (c) in 0.1 M PBS pH 7.0 under N_2_-saturated at a scan rate of 100 mV/s.

The electrochemical behavior of fabricated magnetic glassy carbon electrode (MGCE) was characterized by cyclic voltammetry. Ferricyanide (K_3_Fe(CN)_6_) was used as a redox probe to investigate the electrochemical behaviors of MGCE comparing to the bare commercial glassy carbon electrode (GCE) with equal diameter of 3 mm at the scan rate of 50 mV/s in 0.1 M PBS pH 7.0. Figure 4A, the CVs curve of the bare GCE in curve a showed a pair of well-defined quasi-reversible peaks with slightly lower peak current and larger peak separation potential than MGCE in curve b. Compared to the bare GCE, the redox peak currents of the bare MGCE increased greatly, implying that the MGCE electrochemical property can be applied for biosensors and BFCs. The electrode surfaces of glassy carbon have been examined by microscopy. Figure S2a–c shows microscopic images of unpolished, polished bare MGCE and bare GCE surface, respectively. It can be seen that the unpolished bare MGCE was rough. After applied aluminium oxide particles to give a polished MGCE surface, the electrode surface was smooth without scratches surface.

### Direct electrochemistry of GOD immobilized Fe_3_O_4_-RGO modified electrode

GOD molecules have flavin adenine dinucleotide (FAD) as redox centers deep localization inside the protein structure, thus the DET for GOD is extremely difficult. In order to improve the electron transfer of FAD, Fe_3_O_4_-RGO was applied to immobilize GOD. Figure 4B shows the cyclic voltammograms of bare MGCE in N_2_-saturated PBS at a scan rate of 100 mV/s. No peaks were observed for GOD/MGCE (curve a) and Fe_3_O_4_/GOD/MGCE (curve b). The background current of Fe_3_O_4_/GOD/MGCE was higher than the GOD/MGCE, which can be ascribed to the large surface area of Fe_3_O_4_ showing a distinct electrochemical response. In Fig. 4B, curve c shows redox peak of Fe_3_O_4_-RGO/GOD/MGCE with anodic peak potential (*E*_*pa*_) at −0.438 V and cathodic peak potential (*E*_*pc*_) at −0.475 V. The peak potential separation (*ΔE*_*p*_) is about 37 mV. These results demonstrate the fast DET kinetics of the GOD on the surface of Fe_3_O_4_ on the graphene sheet. The well-defined and quasi-reversible redox peaks suggest favorable direct-electron transfer between the electrode and redox centers of GOD molecules. The formal potential (*E* ^*0′*^) obtained by averaging potential values of the *E*_*pa*_ and *E*_*pc*_ was −0.457 V. This value is close to the standard electrode potential of −0.483 (vs. Ag/AgCl) for FAD/FADH_2_ at pH 7.0^28^, suggesting that the GOD molecules retain bioactivity after adsorption on the Fe_3_O_4_-RGO nanocomposites. The DET process mechanism was described in equation (1) and (2). GOD is two protons and two electrons coupled reaction, FAD serves as the catalytic site of GOD by accepting the electrons donated by the glucose and being reduced to FADH_2_. In this process glucose is converting into gluconolactone. (GOD)FADH_2_ is then oxidized by electrode to (GOD)FAD. Two protons and two electrons can subsequently be transferred from GOD to bioanode. O_2_ is a natural electron acceptor for GOD. In presence of O_2_, GOD can be transfer electron to O_2_ then reduced into hydrogen peroxide as present in equation (3). Therefore, O_2_ is a competing electron acceptor to DET reaction. Unfortunately, it is well know that DET system did not require oxygen due to FAD serves as the catalytic site of GOD by accepting the electrons donated by the glucose and being reduced to FADH_2_. The electrons can be transferred directly from GOD to electrode through Fe_3_O_4_-RGO composite. Moreover, based on membraneless EBC, the cathode compartment could be consumed most of O_2_ for reduction reaction. In addition, the stability of Fe_3_O_4_/GOD/MGCE was also evaluated as shown in Fig. S3. There was no obvious change in redox peaks could be seen from the CV curves, the CVs curves still almost remained from their initial cycle after continuous scanning for 100 scan cycles. This can be implied that the fabricated electrode is very stable.

1 $${\rm{GOD}}({\rm{FAD}})+{\rm{glucose}}\to {\rm{GOD}}({{\rm{FADH}}}_{2})+{\rm{gluconolactone}}$$

2 $${\rm{GOD}}({{\rm{FADH}}}_{2})\leftrightarrow {\rm{GOD}}({\rm{FAD}})+2{{\rm{H}}}^{+}+2{{\rm{e}}}^{-}$$

3 $${\rm{GOD}}({{\rm{FADH}}}_{2})+{{\rm{O}}}_{2}\to {\rm{GOD}}({\rm{FAD}})+{{\rm{H}}}_{2}{{\rm{O}}}_{2}$$

### The influence of scan rate

**Figure 5:**
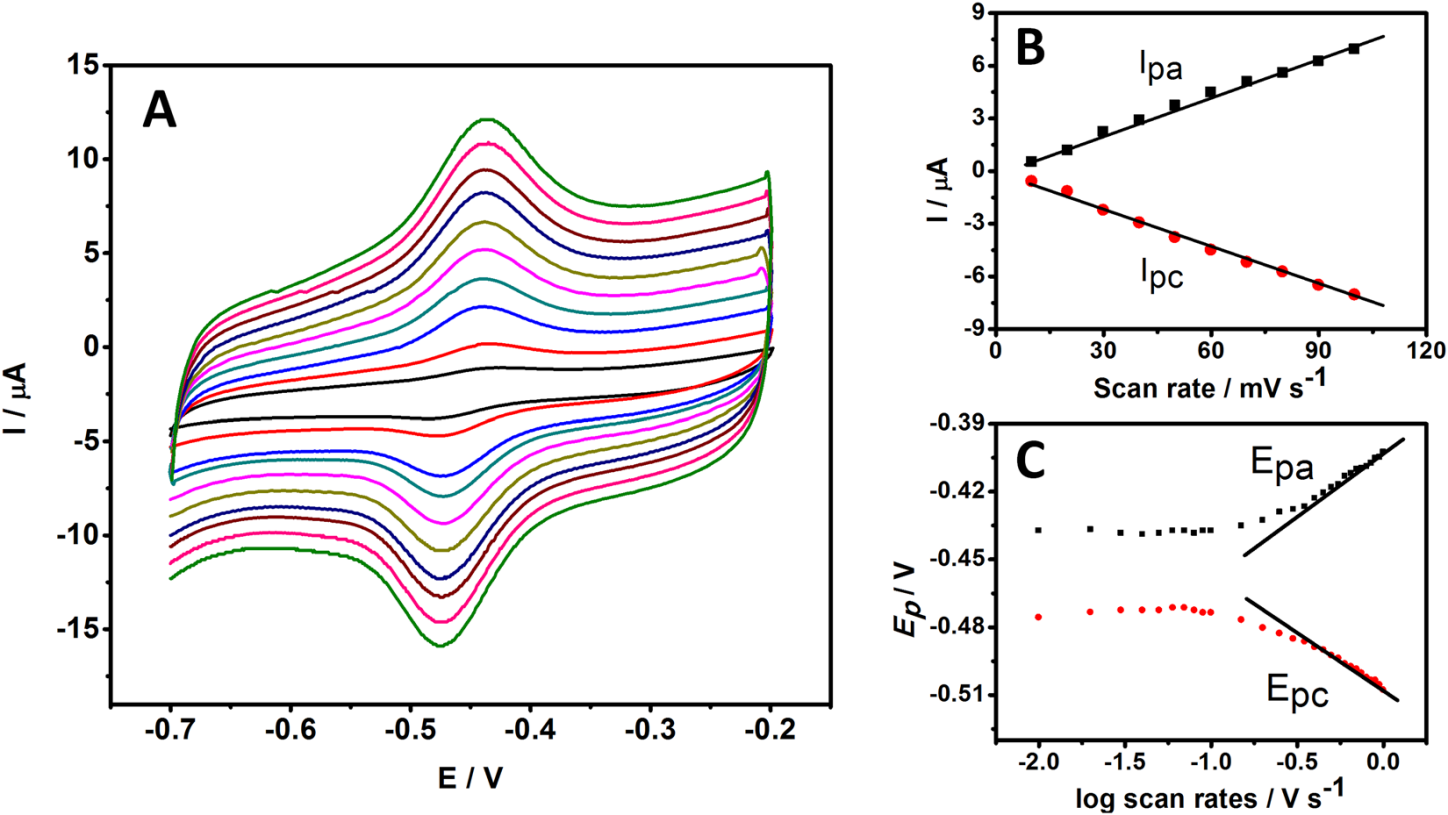
(**A**) CVs of the RGO-Fe_3_O_4_/GOD/MGCE in 0.1 M PBS pH 7.0 at different scan rates inner to outer: 10, 20, 30, 40, 50, 60, 70, 80, 90 and 100 mV/s. (**B**) The plot of the peak current vs. scan rates. (**C**) The relationship of the peak potential vs. the logarithm of scan rate from 10 to 1000 mV/s.

The effect of the scan rate on cyclic voltammetric performance at the Fe_3_O_4_-RGO/GOD modified MGCE is shown in Fig. 5A. The redox processes of nanocomposite gave almost symmetric anodic and cathodic peaks (*E*_pa_ and *E*_pc_) at relatively slow scan rates. When the scan rate increases, the redox potentials of GOD shift slightly. The anodic and cathodic peak currents linearly increased with the increasing scan rate from 10 to 100 mV/s. This indicates that the redox reaction of GOD on Fe_3_O_4_-RGO modified electrode was a quasi-reversible surface-controlled process. The surface concentration (*Γ*, mol/cm^2^) of electroactive GOD can be calculated as 2.03 × 10^−11^ mol/cm^2^ according to the formula *Γ* = Q/n*FA*, where *Q* is the charge consumed in *C*, n is the number of electrons transferred (*n* = 2), *A* is the electrode area (cm^2^) and *F* is the faraday constant. Figure 5B displays the plot of cathodic peak current versus the scan rate, a linear relationship with linear regression equations: *I*_*pa*_ = 0.071x − 0.0337, *I*_*pc*_ = −0.073x + 0.0384 with a correlation coefficient of 0.994 and 0.996, respectively. These results demonstrate the fast DET kinetics of the GOD on the surface of graphene. According to Laviron’s equation^29^, the plots of *E*_pa_ and *E*_pc_ vs. the logarithm of the scan rates (log *ν*) produce two straight lines with slopes of 2.3RT/(1−*α*)nF and −2.3RT/*α*nF at high scan rates, as shown in Fig. 5C. The charge transfer coefficient (*α*) and the electron transfer rate constant (*k*_s_) for the proposed electrode were calculated as 0.5 and 20.07 s^−1^, respectively, according to following Equation 4. This result indicated that RGO-Fe_3_O_4_ facilitates fast electron transfer between the redox-active site of enzymes and the surface of electrode. The immobilized GOD on RGO-Fe_3_O_4_ electrode provides fast electron transfer between the redox center of the enzyme and the surface of the electrode, implying that graphene can remarkably enhance the DET kinetics of GOD.

4 $$\mathrm{log}\,{k}_{s}=\alpha \,\mathrm{log}(1-\alpha )+(1-\alpha )\mathrm{log}\,\alpha -\,\mathrm{log}\,\frac{RT}{nFv}-\frac{\alpha (1-\alpha )nF{\rm{\Delta }}{E}_{p}}{2.3RT}$$

### Electrocatalytic behavior of the Fe_3_O_4_-RGO/GOD/MGCE

**Figure 6:**
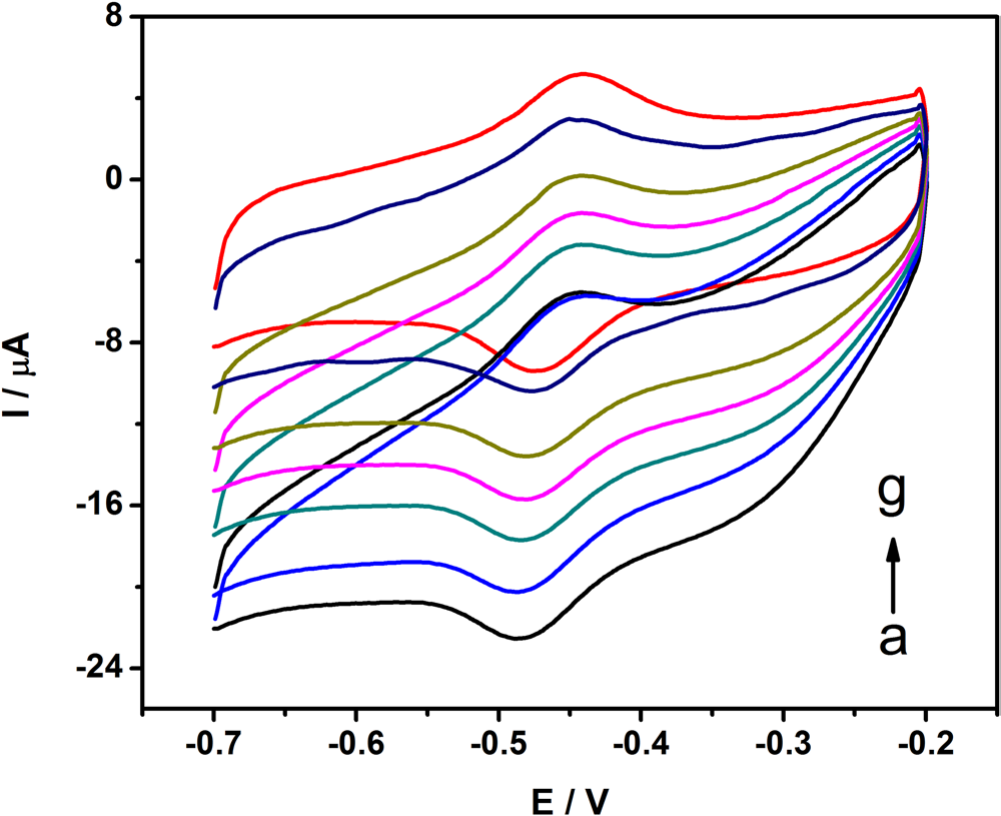
CVs of Fe_3_O_4_-RGO/GOD/MGCE in 0.1 M PBS pH 7.0 at a scan rate of 50 mV/s in the presence of different concentrations of glucose O_2_-saturated without glucose (a), with glucose concentration of 0.5, 1, 2, 4 and 8 mM (b–f) and N_2_-saturated without glucose (g).

Figure 6 shows the CVs of the Fe_3_O_4_-RGO/GOD/MGCE in a solution containing different concentrations of glucose under the condition of oxygen saturation. It can be seen from this figure that the reduction decreased with an increase in glucose concentration ranging from 0.5 mM to 8 mM. It can be explained that glucose is the substrate of GOD. When added to the air-saturated PBS, the enzyme-catalyzed reaction occurs and the concentration of the oxidized form of GOD present as GOD(FAD) at electrode decreases. Thus, the addition of glucose restrained the electrocatalytic reaction and led to the decrease of the reduction current. Therefore, this nanocomposite can serve as an efficient glucose sensor and EBC.

### Electrocatalytic behavior of biocathodes

**Figure 7:**
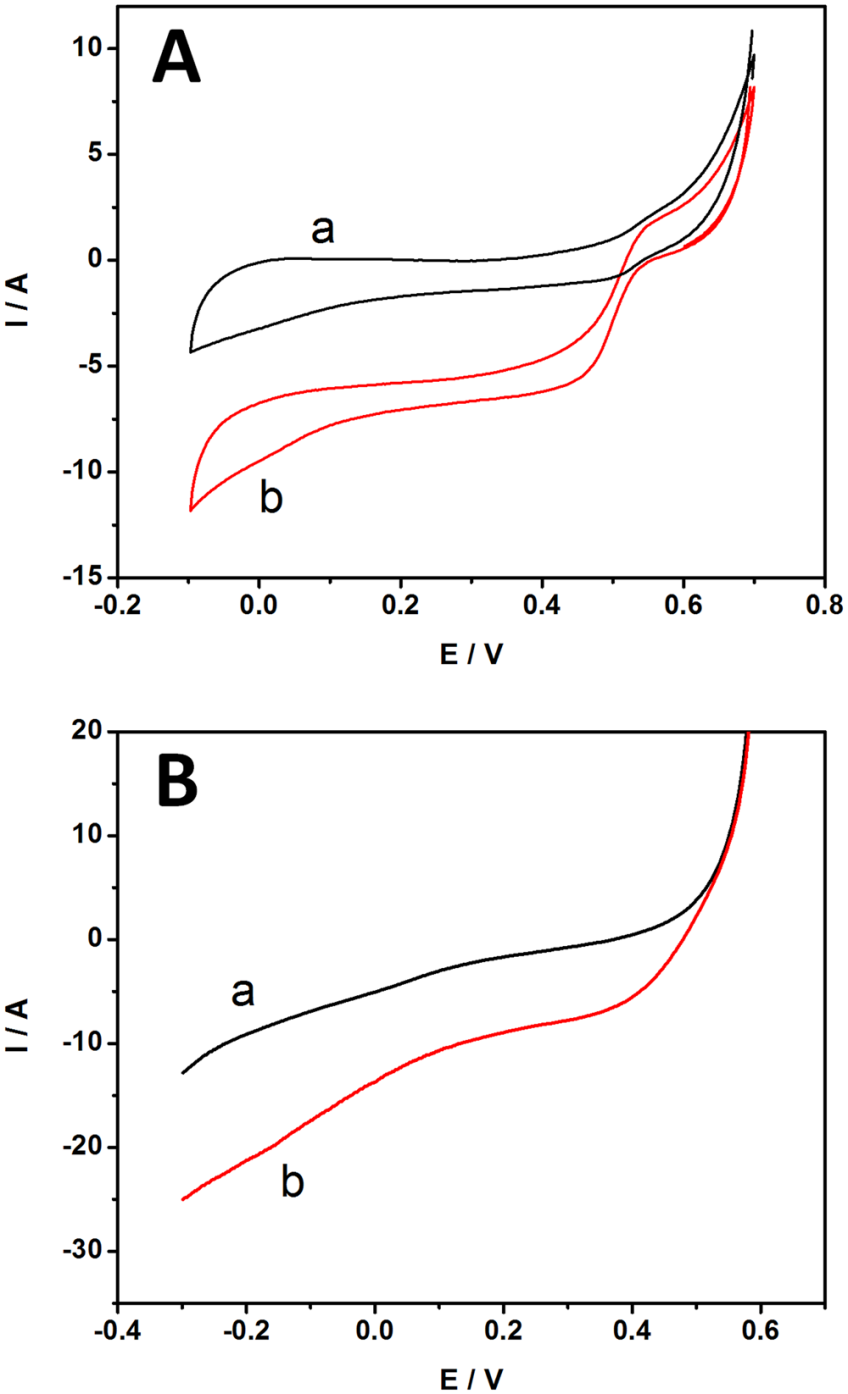
(**A**) CVs of Fe_3_O_4_-RGO/BOD/MGCE under N_2_ (a), and O_2_ (b) saturated 0.1 M PBS pH 7.0 at a scan rate of 1 mV/s. (**B**) LSVs of Fe_3_O_4_-RGO/BOD/MGCE under N_2_ (a) and O_2_ (b) saturated in 0.1 M PBS pH 7.0 at a scan rate of 1 mV/s.

For biocathodes, the reduction of O_2_ generally utilized two types of enzymes, including bilirubin oxidase and laccase. Laccase presents an optimum activity around pH 4-5^30^. BOD electrocatalytic activity was investigated at neutral pH or pH 7.0 which was suitable for a real application system. BOD is one of the multicopper oxidase selected for catalyzing the four-electron reduction of oxygen to water at the biocathode because BOD can efficiently work as an electrode biocatalyst even under neutral conditions. In their structure, the T1 copper site gives electrons to the electrode and transfers those electrons to the T2/T3 copper site^31^, where oxygen is reduced to water in a four-electron transfer mechanism according to Equation 5. The electrocatalytic reaction of BOD on Fe_3_O_4_-RGO/MGCE as biocathode was examined using CV. The experiments were carried out under nitrogen saturated and oxygen saturated conditions at the potential between −0.1 V to + 0.7 V at a scan rate of 1 mV/s. Figure 7A displays the CVs recorded at Fe_3_O_4_-RGO/BOD/MGCE in N_2_ (curve a) and O_2_ (curve c) saturated 0.1 PBS pH 7.0. It can be seen that the presence of oxygen in the system and a highly enhanced cathodic peak current increase was observed with a peak potential of oxygen reduction of +0.51 V versus Ag/AgCl. The potential of the peak begins at +0.60 V, whereas the presence of nitrogen in the modified electrodes exhibited no catalytic activity. These results agree with literature data of approximately +0.5 V (vs. Ag/AgCl)^32^, which is close to the redox potential of the T1 copper site BOD. It demonstrates that the modified electrode that immobilized BOD has the capability to achieve DET and efficiently catalyze oxygen reduction to water. Linear sweep voltammetry (LSV) was also used to study the electrocatalytics of RGO-Fe_3_O_4_/BOD/MGCE. Figure 6B shows the linear sweep voltammograms (LSVs) obtained at RGO-Fe_3_O_4_/BOD/MGCE in oxygen saturated 0.1 M PBS pH 7. The reduction current increased, indicating that oxygen reduction activity occurred at the RGO-Fe_3_O_4_/GOD/MGCE. As shown in Fig. 7B in curve b, O_2_ saturated the current response and showed significant increase by two-fold compared to N_2_ saturated (curve a). The biocatalytic appears potential at +0.51 V, indicating BOD was envisaged as the new biocathode of EBC for oxygen reduction in a neutral medium.

5 $${{\rm{O}}}_{2}+4{{\rm{H}}}^{+}+4{{\rm{e}}}^{-}\to 2{{\rm{H}}}_{2}{\rm{O}}$$

### The performance of EBC

**Figure 8:**
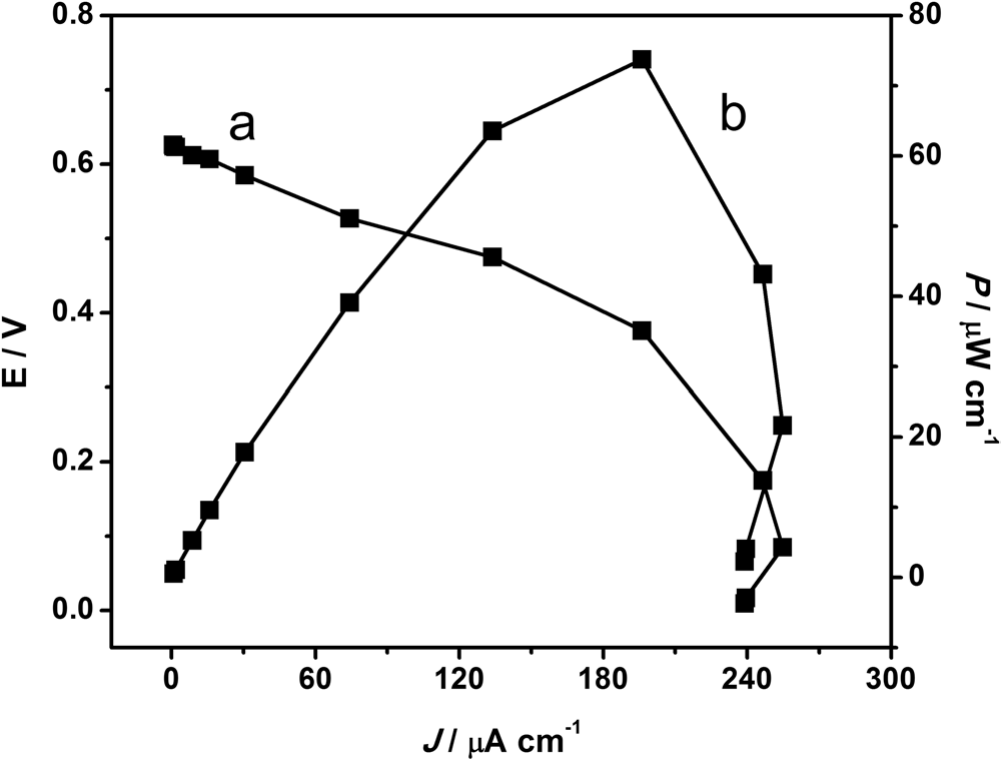
Polarization curve (a) and power density curve (b) of the mediatorless based glucose EBC on current density in 0.1 M PBS pH 7.0 containing 5 mM glucose under O_2_-saturation.

EBC performance was measured with RGO-Fe_3_O_4_/GOD/MGCE as the bioanode and RGO-Fe_3_O_4_/BOD/MGCE as the biocathode without a separating membrane, as shown in Fig. 1. The EBC system was operated at 37 °C in 0.1 M PBS pH 7.0 and 5 mM of glucose was used as fuel. At the bioanode, glucose was oxidized by GOD to gluconolactone, where the electrons were transferred from the GOD to the Fe_3_O_4_-RGO/MGCE. Electrons were flowed through an external circuit then released at the biocathode to BOD, where oxygen was reduced into water. An electrical current is generated as a result of the electrons flow. The cell voltage was measured with a multi-meter under different loads varying from 10 MΩ to 200 Ω applied to the EBC system. The current and power density was calculated from the voltage using ohm’s law. Figure 8 shows two curves of the polarization curve and power density curves of the EBC in the presence of 5 mM glucose, describing the dependence of both open circuit voltage (OCV) and the power density (*P*) on current density (*j*) of the bioelectrode based glucose EBC. The OCV of the EBC was around +0.626 V, the maximum current density was 380 µA cm^−2^ and the maximum power density was 73.7 µW cm^−2^ at +0.38 V which was higher than the glucose EBC reported of V. Krikstolaityte *et al*., 2013 (3.5 µW cm^−2^)^33–38^ and A. Ramanavicius *et al*., 2015 (4.2 µW cm^−2^)^39^. The improvement of power output can be achieved by Fe_3_O_4_-RGO nanocomposite. The performance of this present glucose EBC is quite comparable and better than some previously reported in literature for mediatorless glucose EBC based on DET in both bioelectrodes, as shown in Table 1. The EBC showed repeatability with an R.S.D of 5.73% for 5 repeatable measurements carried out with the flow system. These results indicate that Fe_3_O_4_-RGO based nanocomposites can be useful materials for the fabrication of EBC to gain energy from biological fuels such as glucose. Moreover, Fe_3_O_4_-RGO has great potential for the fabrication of glucose EBC due to operation in the physiological conditions of humans, preparation protocols and simple EBC assembly protocols without mediators or membranes.

**Table 1:** Comparison of glucose EBC based on mediatorless type for both cathode and anode.

Modified electrode	Anode enzyme	Cathode enzyme	OCV (V)	Power density (μW cm^−2^)	Glucose concentration	Ref.
SWNT/pSi	GOD	laccase	—	1.38	4 mM, pH 7	33
SWNT/PPR: CNP/PPR	GOD	tyrosinase	—	157.4	1 mM, pH 6.5	17
CNT disks	GOD/catalase	laccase	0.95	1300	5 mM, pH 7	11
CNTs-IL/CP	GDH	BOD	0.56	13.5	30 mM, pH 7	34
NPNW	GOD	laccase	0.23	30	—	35
Graphene/SWNT cogel	GOD	BOD	0.61	190	100 mM, pH 7	36
CNDs/GC	GOD	BOD	0.93	40.8	4 mM, pH 7.2	32
CNT-PEI	GOD	laccase	—	102	40 mM, pH 5.5	37
Fe_3_O_4_-RGO/MGCE	GOD	BOD	0.63	73.7	5 mM, pH 7	This work

CNDs: carbon nanodots; pSi: porous silicon wafer; SWNTs: SWNTs; GOD: glucose oxidase; BOD: bilirubin oxidase; SPGE: spectrographic graphite electrodes; GDH: Glucose dehydrogenase; CNTs-IL: ionic liquid functionalized carbon nanotubes; NPNW: Nafion/poly(vinyl pyrrolidone) compound nanowire; CP: carbon paper; CG: carbon aerogel; CNP: carbon nanopowder; PEI: Poly(ethylenimine); PPR: polypyrrole.

### The stability of EBC

The stability of EBC was characterized by measuring its power loss when continuously working in an air-saturated quiescent buffer containing 5 mM glucose coupling with 1 MΩ of resistance loaded on the cell. The maximum power density was observed for 4 weeks. After operating for 24 h, the power of the EBC retained 98.37 % of its original power output and held steady at 95.39 % after duration of 7 days. Then 78.7 % of initial power density was retained even after 4 weeks, which revealed good durability and stability of the fabricated EBC as shown in Fig. S4. However, the OCV of the EBC remained unchanged during the duration. This could indicate that covalent bonding was unaffected by changes in the surrounding environment. Moreover, the electrostatic interaction binds with enzymes and magnetic force at MGCE to prevent leaching from the electrode surface during operation and storage. In EBC development, the stability of the enzymes on the electrode is the main factor for retaining long-term performance of the membraneless EBC. However, no significant breakthrough has been achieved in this work concerning the longevity of EBCs.

## Methods

### Reagents and materials

Graphene oxide (GO > 99 wt% purity and total thickness < 3 nm. Average dimensions of individual flakes ranged from 300–800 nm, Glucose oxidase (EC 1.1.3.4 from *Aspergillus niger*), Bilirubin Oxidase (EC 1.3.3.5 from *Myrothecium verrucaria*), Iron (III) chloride hexahydrate (FeCl_3_.6H_2_O), 1-ethyl-3-(3-dimethyaminopropyl) carbodiimide (EDC),N-hydroxysuccinimide (NHS), Ammonium hydroxide (NH_4_OH), 1,6-Hexanediamine, Glucose, Sodium acetate (NaAc) and Ethylene glycol (EG) were purchased from Sigma Aldrich and used without further purification. A 0.1 M phosphate buffer saline PBS, pH 7.0 was prepared by mixing solutions of Na_2_HPO_4_ and NaH_2_PO_4_. The glucose solution was prepared overnight before use to allow mutarotation for 24 hours. All solutions were prepared with deionized water (DI).

### Apparatus

The electrochemical experiments were performed with a potentiostat (Metrohm Autolab PGSTAT302N, Ecochemie, Netherlands). The three electrode system was performed with a homemade magnetic glassy carbon electrode (MGCE) as a working electrode, a platinum wire as a counter electrode and an Ag/AgCl saturated KCl as the reference electrode.

### Magnetic glassy carbon electrode fabrication

The magnetic glassy carbon electrode (MGCE) was prepared by placing a nummular NdFeB magnet (3 mm in diameter and 4 mm in thickness) on a glassy carbon (3 mm in diameter and 3 mm in thickness). A copper wire was put around the magnet and filled with silver glue, which was then put into an acrylic tube (10 mm in diameter and 20 mm in depth). All components were fixed with epoxy resin then cured at room temperature for at least 24 h. The MGCE was successively polished with emery paper and alumina powder, followed by sonication in DI water.

### Preparation of Fe_3_O_4_-RGO nanocomposite modified electrode

The NH_2_-Fe_3_O_4_ NPs were prepared using a solvothermal method. Briefly, FeCl3·6H2O 1 g and sodium acetate 3 g were dissolved in 30 mL of ethylene glycol and 6.5 g of 1,6-hexanediamine. After the mixtures were dispersed, the yellow solution was transferred to a teflon-lined stainless-steel autoclave and sealed to heat at 200 °C for 6 h. Then the autoclave was cooled to room temperature. The obtained black magnetite particles were washed with ethanol for several times. Finally the products were dried under vacuum at room temperature. The Fe_3_O_4_ NPs was immobilized on GO using EDC and NHS as coupling agents by the formation of an amide link between the amino group of Fe_3_O_4_ NPs and the carboxyl group of GO. 40 mg GO, 200 mg EDC and 160 mg NHS were added into 60 mL DI water. The mixture was ultrasonicated for 30 min to form a homogenous suspension. Next, 40 mg of NH_2_-Fe_3_O_4_ NPs was added into the suspension solution and the mixture was subjected to ultrasonication for 30 min. The reaction was carried out at 80 °C for 5 h under stirring. The Fe_3_O_4_-GO nanocomposite was obtained by magnetic separation and washed with water several times. Fe_3_O_4_-RGO nanocomposite based on glucose reduction was prepared. In brief, 40 mg glucose was added into a 25 mL Fe_3_O_4_-GO dispersion solution followed by stirring for more than 0.5 h. A 100 µl ammonia solution (25% w/w) was added into the resulting dispersion solution. After that, the mixture was stirred for 60 min at 95 °C. Finally, the resulting stable black dispersion solution was washed with water several times. The obtained Fe_3_O_4_-RGO nanocomposite (10 mg/1 mL) was redispersed in DI water before further use. Finally, the 10 µl Fe_3_O_4_-RGO nanocomposite solution was dropped onto the MGCE surface.

### Bioelectrode fabrication

GOD was immobilized onto the Fe_3_O_4_-RGO modified MGCE through electrostatic interaction between the positive charge of Fe_3_O_4_ and the negative charge of GOD. 30 mg of GOD was added into 1 mL 0.1 M PBS pH 7.0 solutions. The Fe_3_O_4_-RGO modified MGCE was immersed into the GOD solution (30 mg/mL in 0.1 M PBS pH 7.0) then reacted at room temperature for 24 h. The Fe_3_O_4_-RGO/GOD modified surface was washed with DI water to remove the unadsorbed enzyme molecules and allowed to dry at room temperature. The modified electrode was stored at 4 °C when not used. The preparation of biocathode used BOD instead of GOD.

### Biofuel cell set up

The EBC cell constructed with three-dimensional printer (3D printer) using poly(lactic acid), PLA as a material. The EBC cell consists of Fe_3_O_4_-RGO/GOD and Fe_3_O_4_-RGO/BOD as bioanode and biocathode, respectively, with electrode spacing of 0.5 cm. The EBC system was fed with 0.1 M PBS pH 7.0 containing 5 mM of glucose delivered to the electrode chamber using a peristatic pump. The voltage output was measured using a multi-meter with application of external resistance (*R*_ext_) varying from 10 MΩ to 200 Ω. The voltage was used to calculated power (*P*) according to the equation *P* = *IV*/*A*, where *I* is the current, *V* is the voltage and *A* is the area of the electrode. The EBC was operated at human physiological temperature (37 ± 1 °C).

## Conclusions

This paper successfully demonstrates a design and simple platform for construction of an enzymatic biofuel cell based on direct electron transfer (mediatorless) BFC by Fe_3_O_4_-RGO/GOD as the bioanode and Fe_3_O_4_-RGO/BOD as the biocathode. Enzymes were incorporated into Fe_3_O_4_-RGO by strong electrostatic interaction. The properties of graphene and magnetic nanoparticles enhance enzymatic biofuel cells for more efficient conductivity and also increase the immobilization of enzymes and modified bioelectrodes without the binder or adhesive agents that usually block electron transfers at electrode surfaces. Fe_3_O_4_ NPs not only increases the surface area, but also has paramagnetic properties which make them more easily manipulated by an external magnetic field to prevent the leakage of enzymes at electrode surfaces. This bioelectrode fabrication approach could offer promising solutions for generations of new classes of membraneless biofuel cells.

## Electronic supplementary material

Magnetic Nanoparticle-Reduced Graphene Oxide Nanocomposite as a Novel Bioelectrode for Mediatorless-Membraneless Glucose Enzymatic Biofuel Cells
