# Supplementary material for: Magnetic Nanoparticle-Reduced Graphene Oxide Nanocomposite as a Novel Bioelectrode for Mediatorless-Membraneless Glucose Enzymatic Biofuel Cells
Source: Sci Rep. 2017 Oct 10;7:12882. doi: 10.1038/s41598-017-12417-0 (PMC5635112; doi:10.1038/s41598-017-12417-0)
Supplement: Supplementary file 1 — Magnetic Nanoparticle-Reduced Graphene Oxide Nanocomposite as a Novel Bioelectrode for Mediatorless-Membraneless Glucose Enzymatic Biofuel Cells [file 41598_2017_12417_MOESM1_ESM.pdf]

Supporting Information

Magnetic Nanoparticle-Reduced Graphene Oxide Nanocomposite as a Novel Bioelectrode for Mediatorless-Membraneless Glucose Enzymatic Biofuel Cells

Saithip Pakapongpan[1,2], Adisorn Tuantranont[2] and Rungtiva P.Poo-arporn[1,*]

[1] Biological Engineering Program, Faculty of Engineering, King Mongkut's University of Technology Thonburi, Bangkok 10140, Thailand

[2] Thailand Organic and Printed Electronics Innovation Center, National Electronics and Computer Technology Center, NSTDA, Pathum Thani 12120, Thailand
E-mail: rungtiva.pal@kmutt.ac.th

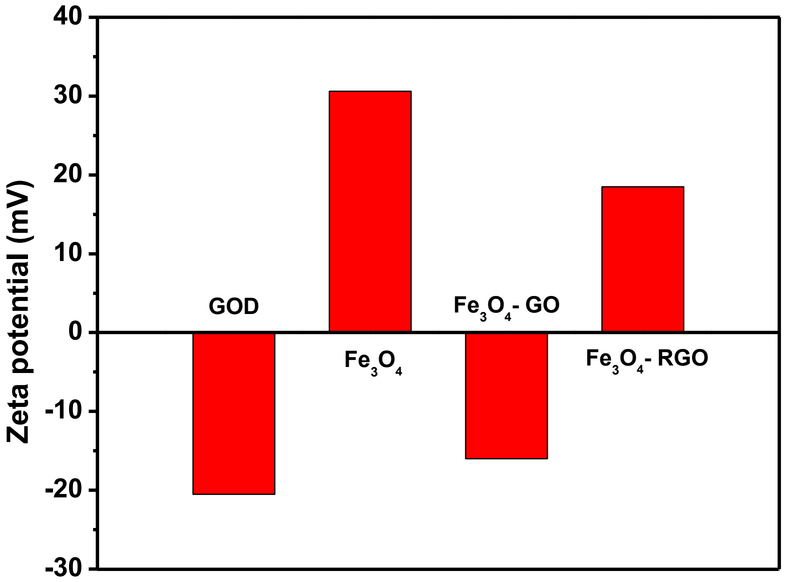

Figure S1. Zeta potentials of GOD, Fe_3O_4, Fe_3O_4-GO and Fe_3O_4-RGO.

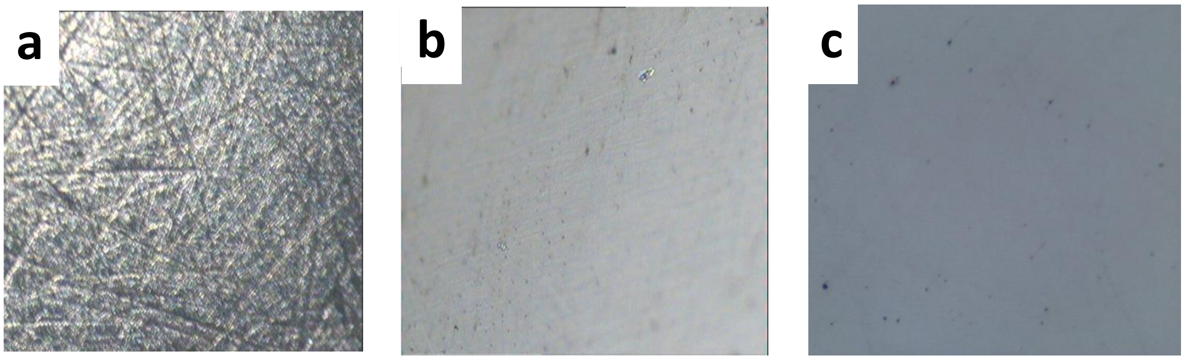

Figure S2. Microscopic images of unpolished (a), polished bare MGCE (b) and GCE commercial (c).

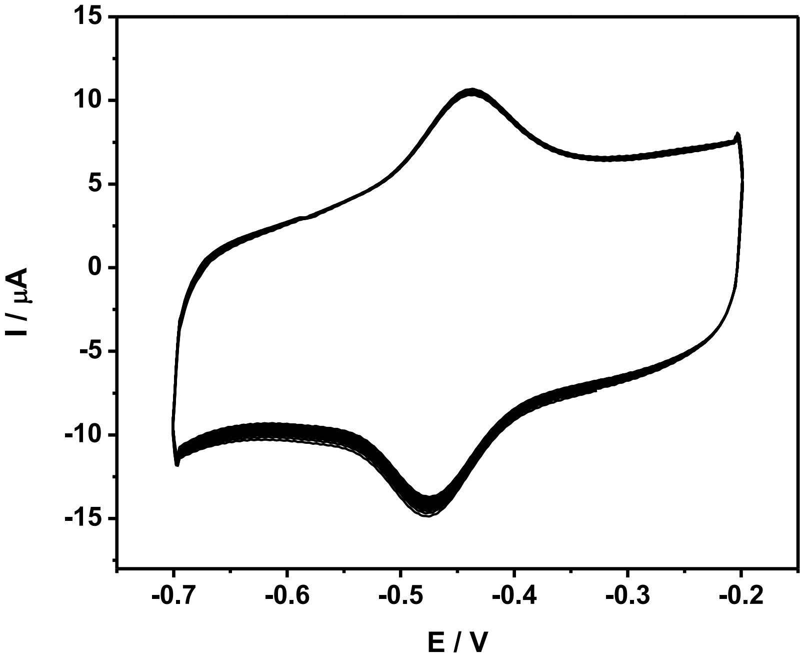

Figure S3. CVs of the Fe_3O_4-RGO/GOD/MGCE on scanning 100 cycles in 0.1 M PBS pH 7.0 under N_2-saturated at a scan rate of 100 mV/s.

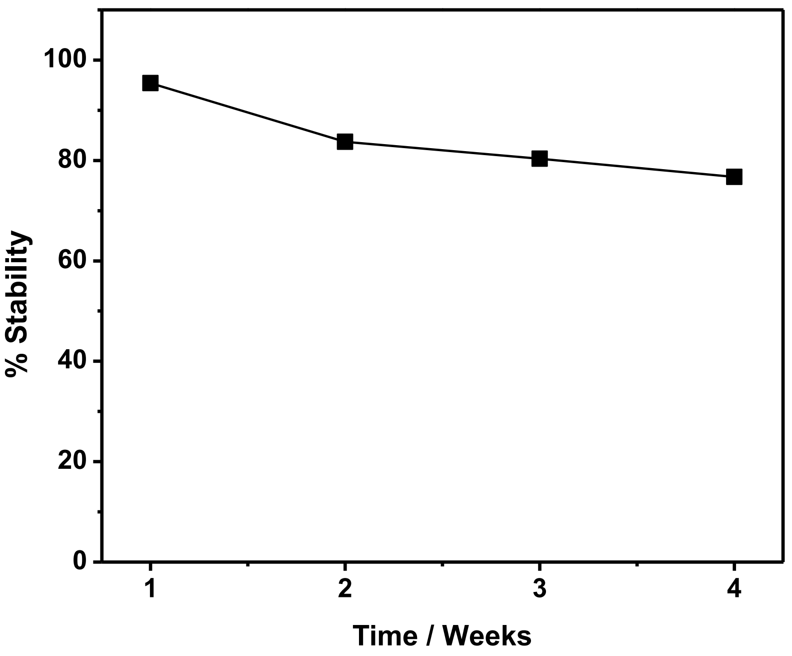

Figure S4. The stability of the DET-based glucose EBC in 0.1 M PBS pH 7.0 containing 5 mM glucose under O_2-saturated.
